# Epoxidation Kinetics of High-Linolenic Triglyceride Catalyzed by Solid Acidic-Ion Exchange Resin

**DOI:** 10.1038/s41598-019-45458-8

**Published:** 2019-06-20

**Authors:** Adhimoolam Bakthavachalam Kousaalya, Shiferaw D. Beyene, Beshah Ayalew, Srikanth Pilla

**Affiliations:** 1 Department of Automotive Engineering, Clemson University, Greenville, SC USA; 2 Clemson Composites Center, Clemson University, Greenville, SC USA; 3 Department of Materials Science and Engineering, Clemson University, Clemson, SC USA; 4 Department of Mechanical Engineering, Clemson University, Clemson, SC USA

**Keywords:** Heterogeneous catalysis, Composites

## Abstract

Epoxidation of high-linolenic perilla oil was carried out in the presence of solid acidic ion-exchange resin at varying reaction temperatures for 8 h. A pseudo two-phase kinetic model that captures the differences in reactivity of double bonds at various positions in the fatty acid of a triglyceride molecule during both epoxy formation and cleavage was developed. The proposed model is based on the Langmuir-Hinshelwood-Hougen-Watson (L-H-H-W) postulates and considers the adsorption of formic acid on the catalyst as the rate-determining step. To estimate the kinetic rate constants of various reactions, genetic algorithm was used to fit experimentally obtained iodine and epoxy values of epoxidized perilla oil. A re-parametrized form of Arrhenius equation was used in the proposed model to facilitate the precise estimation of parameters with least computational effort. The obtainment of the least error between experimentally determined and theoretically predicted iodine and epoxy values indicates the robustness of the proposed model.

## Introduction

A plethora of research initiatives have been undertaken in recent years towards the synthesis of Bisphenol-A-free epoxies derived from biological sources as alternatives to Diglycidyl ether of bisphenol-A^1^. Unfortunately, these initiatives have consistently neglected the need to replace epichlorohydrin – a Class-1b carcinogen. This has resulted in triglycerides (such as vegetable oils) remaining the only source for obtaining sustainable non-toxic epoxies (i.e., epoxidized vegetable oils or EVOs)^2,3^ till date. Regrettably, such epoxies are characterized by poor mechanical properties due to the presence of fewer chemically modifiable groups, resulting in lower crosslinking density^4^. This can be tackled by the appropriate selection of highly unsaturated triglycerides that may result in increased number of epoxy groups in EVOs. However, obtaining such increased number of epoxy groups may prove challenging, as there exists the possibility of steric hindrance and electronic effects from the bulkier glycerol center (in triglycerides) influencing the reactivity of double bonds present at varying positions in fatty acids^5^. Further, the most commonly employed epoxidation reaction for triglycerides – namely, the Prilezhaev reaction – is accompanied with several side reactions (including simultaneous oxirane cleavage) that are significantly influenced by their reaction conditions. Hence, selection of ideal reaction conditions and synthesis procedure, along with the knowledge of steric/electronic effects on reactivity of functional groups (via epoxidation kinetics) is imperative for obtaining triglycerides with higher number of epoxy groups^6^.

In this regard, epoxidation of triglycerides via Prilezhaev reaction has been widely studied in the presence of inorganic acid as catalyst, but is often critiqued for the role of the acid in causing extensive oxirane cleavage during the reaction^7^. This has led to a shift towards solid acidic ion-exchange resins (AIER) as alternatives to liquid inorganic acids^8^. AIER possesses the ability to trap various reactants that can react with epoxy, such as formic acid and hydrogen peroxide, resulting in EVOs with minimal oxirane cleavage^8–10^. However, such a shift is marked by enhanced complexity in epoxidation reaction kinetics due to chemical reactions occurring in three phases (oil, water and solid catalyst)^11^.

A number of studies have attempted to understand this complexity in kinetics of epoxidation reaction via modelling. In this regard, the first study was carried out by Wisniak and Navarrete^12^ for triglyceride obtained from fish oil, but their model failed to adequately explain reaction kinetics due to its simplistic assumption of the reaction being homogeneous^13,14^. Since then, numerous kinetic models have been proposed^6,9,11,14,15^ that can be classified^16^ mainly into pseudo-homogeneous (P-H)^14^ and pseudo two-phase (P2P)^11^ models. The P-H model^14^ accounts solely for the reaction occurring between both liquid phases (oil and water), while assuming that solid AIER dissolves completely and acts only as a source of protons^17^. In contrast, the P2P model^11^ considers the reactions occurring between solid AIER and liquid phases, and is developed on the basis of Langmuir-Hinshelwood-Hougen-Watson (L-H-H-W) postulates. The P2P model is observed as being better in predicting epoxidation kinetics of heterogeneously catalyzed reactions when compared with the P-H model^6^.

Regrettably, all these models have focused only on high-oleic (C18:1) vegetable oils (i.e., containing double bond only at the 9^th^ position), thereby ignoring the effect of glycerol center on the reactivity of double bonds present in different positions in the fatty acids of a triglyceride molecule^5,18^. This becomes critical, given that Janković *et al*.^18^ have reported that a modified version of P-H model which accounts for fatty acid composition is more accurate in predicting epoxidation kinetics of soybean oil containing 10% linolenic acid (C18:3). However, this model^18^ does not dwell into details on the extent of accuracy and reasons for its higher accuracy upon taking into account the composition of constituent fatty acids.

Therefore, this paper details a P2P model that considers variation in reactivity of double bond due to the effect of glycerol center based on its position during the epoxidation reaction accounting for both epoxy formation and cleavage (via attack by formic acid). High-linolenic perilla oil (containing >65 wt.% linolenic acid) was epoxidized via Prilezhaev reaction^19^. Reaction kinetics was constantly monitored by experimentally determining the variation in iodine and epoxy values at regular time intervals. Various reaction kinetic parameters were determined by fitting experimentally obtained data using genetic algorithm that was selected given the flexibility and simplicity it offers in optimization^20^. Robustness of the proposed model was ascertained by obtaining the least error between experimentally obtained results and model-predicted values. The outcome of this work can enable the selection of optimal synthesis conditions for triglycerides with high unsaturation content that will result in EVOs superior to conventional DGEBA for futuristic applications.

## Kinetic Model

**Figure 1:**
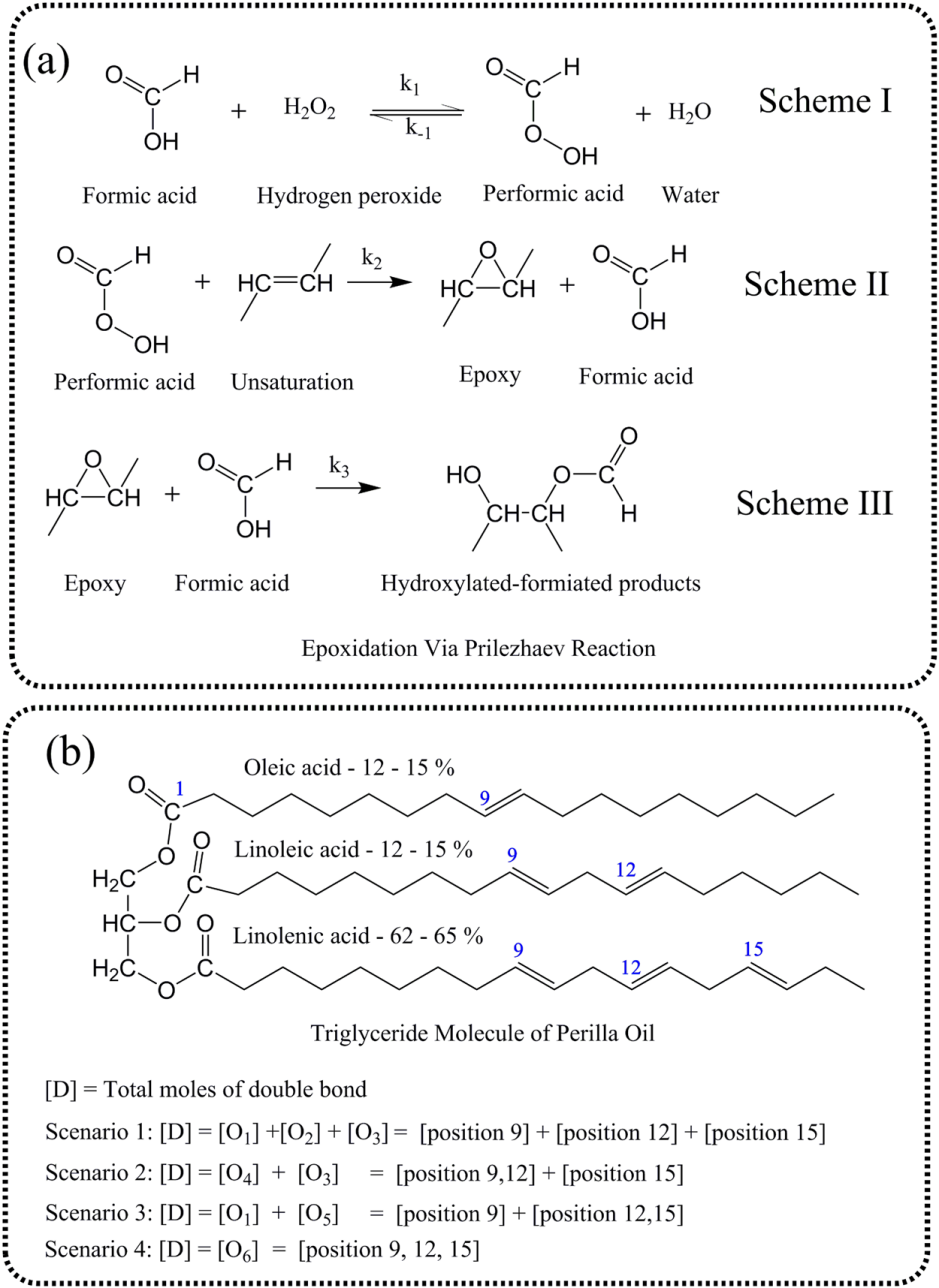
(**a**) Major reactions occurring during the epoxidation of triglyceride: (i) Reaction I: Acid-catalyzed formation of performic acid; (ii) Reaction II: Formation of epoxy groups via reaction between performic acid and double bond; and (iii) Reaction III: Ring-opening reaction due to attack of formic acid on epoxy groups; and (**b**) Triglyceride molecule that indicates the position of double bonds in different fatty acids.

Epoxidation of triglycerides via Prilezhaev reaction is a two-step process: (a) Performic acid formation (via acid catalysis, Scheme I, Fig. 1a), and (b) Epoxidation reaction (Scheme II, Fig. 1a)^21^. These two steps are accompanied by the simultaneous occurrence of multiple side reactions such as the cleavage of epoxy rings and decomposition of reactant molecules. Among these side reactions, the most predominant is epoxy cleavage due to the attack of formic acid, resulting in the formation of hydroxylated-formiated products (Scheme III, Fig. 1a)^11^. The generalized reaction kinetics of these reactions (shown in Fig. 1a) is expressed by multiple first-order differential equations (Eqs (1–4)), assuming negligible mass and heat transfer resistance due to intense stirring.

1 $$\frac{d[F]}{dt}=\frac{d[H]}{dt}+\frac{d[E]}{dt}$$

2 $$\frac{d[P]}{dt}=\frac{-d[H]}{dt}+\frac{d[D]}{dt}$$

3 $$\frac{d[W]}{dt}=\frac{-d[H]}{dt}$$

4 $$\frac{d[HA]}{dt}=-(\frac{d[E]}{dt}+\frac{d[D]}{dt})$$

here, square bracket “[]” denotes the concentration of a particular chemical species (in moles per 100 g of oil). Thus, formic acid [*F*], performic acid [*P*], water [*W*], hydrogen peroxide [*H*], hydroxyl acetate [*HA*], double bond [*D*] and epoxy [*E*] are denoted respectively.

However, to understand the effect of glycerol center on the reactivity of double bond present at different positions, four scenarios (S1, S2, S3 and S4) were assessed in our proposed model (Fig. 1b). These four scenarios assume that the effect of glycerol center on reactivity of any functional group (i.e., double bond and epoxy) in a given position is the same. However, the reactivity of the functional group $(\frac{d[D]}{dt})$ and $(\frac{d[E]}{dt})$ at different positions (either 9^th^ or 12^th^ or 15^th^ position) is considered based on mathematical permutations, as explained below. The kinetic equations corresponding to these different permutations for both $(\frac{d[D]}{dt})$ and $(\frac{d[E]}{dt})$ are expanded below for different scenarios (S1, S2, S3 and S4) based on the assumed extent of reactivity:

- Scenario 1 (S1) considers the reactivity of double bonds to be highly influenced by their position, i.e., double bonds at various positions exhibit differences in their reactivity. Hence:
  $$\frac{d[D]}{dt}=-\,{k}_{{2}_{a}}[P][{O}_{1}]-{k}_{{2}_{b}}[P][{O}_{2}]-{k}_{{2}_{c}}[P][{O}_{3}]$$
  $$\begin{array}{lll}\frac{d[E]}{dt} & = & \{{k}_{{2}_{a}}[P][{O}_{1}]-{k}_{{3}_{a}}[E{O}_{1}]{[F]}^{2}\}+\{{k}_{{2}_{b}}[P][{O}_{2}]-{k}_{{3}_{b}}[E{O}_{2}]{[F]}^{2}\}\\  &  & +\,\{{k}_{{2}_{c}}[P][{O}_{3}]-{k}_{{3}_{c}}[E{O}_{3}]{[F]}^{2}\}\end{array}$$
- Scenario 2 (S2) considers the reactivity of double bonds at positions 9 and 12 to be equal, while assuming the reactivity of double bond at the 15^th^ position to be different from those at the 9^th^ and 12^th^ positions. Hence:
  $$\frac{d[D]}{dt}=-\,{k}_{{2}_{d}}[P][{O}_{4}]-{k}_{{2}_{c}}[P][{O}_{3}]$$
  $$\frac{d[E]}{dt}=\{{k}_{{2}_{d}}[P][{O}_{4}]-{k}_{{3}_{d}}[E{O}_{4}]{[F]}^{2}\}+\{{k}_{{2}_{c}}[P][{O}_{3}]-{k}_{{3}_{c}}[E{O}_{3}]{[F]}^{2}\}$$
- Scenario 3 (S3) considers the reactivity of double bonds at positions 12 and 15 to be the same, and that of double bond at the 9^th^ position to be different from those at the 12^th^ and 15^th^ positions. Hence:
  $$\frac{d[D]}{dt}=-\,{k}_{{2}_{a}}[P][{O}_{1}]-{k}_{{2}_{e}}[P][{O}_{5}]$$
  $$\frac{d[E]}{dt}=\,\{{k}_{{2}_{a}}[P][{O}_{1}]-\,{k}_{{3}_{a}}[E{O}_{1}]{[F]}^{2}\}+\{{k}_{{2}_{e}}[P][{O}_{5}]-\,{k}_{{3}_{e}}[E{O}_{5}]{[F]}^{2}\}$$
- Scenario 4 (S4) considers the reactivity of double bonds at all positions (9, 12 and 15) to be equal. Hence:

$$\frac{d[D]}{dt}=-\,{k}_{{2}_{f}}[P][{O}_{6}]$$

$$\frac{d[E]}{dt}=\{{k}_{{2}_{f}}[P][{O}_{6}]-{k}_{{3}_{f}}[E{O}_{6}]{[F]}^{2}\}$$

here, ${k}_{{2}_{a}},\,{k}_{{2}_{b}},\,\,{k}_{{2}_{c}},\,{k}_{{2}_{d}},\,{k}_{{2}_{e}},\,\,{k}_{{2}_{f}}$ are the rate constants that correspond to epoxy formation, while ${k}_{{3}_{a}},\,{k}_{{3}_{b}},\,{k}_{{3}_{c}}\,{k}_{{3}_{d}},\,{k}_{{3}_{e}},\,{k}_{{3}_{f}}$ are the rate constants that correspond to epoxy cleavage reaction, at different bond positions for different scenarios.

The following initial conditions (@ time, t = 0) were used in model computations (where total double bond concentration [*D*] is 0.77 moles/100 g of oil):

$${\rm{Scenario}}\,{\rm{S}}1:[D]=[{O}_{1}]+[{O}_{2}]+[{O}_{3}]=46.5 \% \,{\rm{of}}\,[D]+30.7 \% \,{\rm{of}}\,[D]+22.8 \% \,{\rm{of}}\,[D]$$

$${\rm{Scenario}}\,{\rm{S}}2:[D]=[{O}_{4}]+[{O}_{3}]=77.2 \% \,{\rm{of}}\,[D]+22.8 \% \,{\rm{of}}\,[D]$$

$${\rm{Scenario}}\,{\rm{S}}3:[D]=[{O}_{1}]+[{O}_{5}]=46.5 \% \,{\rm{of}}\,[D]+53.5 \% \,{\rm{of}}\,[D]$$

$${\rm{Scenario}}\,{\rm{S}}4:[D]=[{O}_{6}]=100 \% \,{\rm{of}}\,[D]$$

Among the three reactions mentioned in Fig. 1a, formation of performic acid (Scheme I) occurs in three steps amidst the presence of AIER catalyst: (a) Adsorption of chemicals onto the surface of solid catalyst; (b) Reaction between these adsorbed chemicals on the catalytic surface; and (c) Desorption of products from the surface of the catalyst^6,9^. Also, unlike homogeneous catalyst-based reactions where performic acid formation is the rate-determining step, either of these three steps (adsorption, surface reaction or desorption) can be the rate-determining step in case of heterogeneous catalyst-based reaction^6^.

Hence, the kinetic rate equation for such a complex reaction (performic acid formation) was developed based on the L-H-H-W postulates. The general form of this equation is shown in Eq. (5), where *k* is kinetic rate constant, *KF* is kinetic factor, *DF* is driving force group, and *A*_*G*_ is the adsorption group. While the rate coefficient of rate-determining reaction is included in *KF*, displacement from chemical equilibrium is explained by *DF*. Further, variation in the rate of reaction is explained by variation in the number of active catalytic sites.

5 $$k=\frac{KF\ast DF}{{A}_{G}}$$

Hence, it is assumed that during chemisorption mechanism, all chemicals in the aqueous phase (formic and performic acids, hydrogen peroxide, and water) were adsorbed onto the surface of the catalyst. Among these chemicals, adsorption of formic acid was assumed to be the rate-determining step for reasons explained by Janković *et al*.^6^ Based on these assumptions and the L-H-H-W postulates, rate of consumption of hydrogen peroxide during the formation of performic acid was obtained from Eq. (6)^6^, where, *M* is the mass of catalyst (in grams), and *C*_*s*_ is the number of moles of active catalytic sites per gram of catalyst.

6 $$\frac{d[H]}{dt}=\frac{-\{M{C}_{s}{k}_{a,F}([F]-\frac{[P][W]}{{K}_{1}[H]})\}}{1+\frac{{K}_{F}[P][W]}{{K}_{1}[H]}+\,{K}_{H}[H]+{K}_{P}[P]+{K}_{w}[W]}$$

here, *K*_*F*_, *K*_*H*_, *K*_*p*_, *K*_w_ are the respective adsorption equilibrium constants for formic acid, hydrogen peroxide, performic acid and water, while *k*_*a*,F_ refers to the adsorption rate constant for formic acid. *K*_1_ refers to the chemical equilibrium constant for performic acid formation, and is obtained from Eq. (7) as described by Santacesaria *et al*.^22^.

7 $${K}_{1}=1.6\exp [\frac{-10000}{R}(\frac{1}{298}-\frac{1}{T})]$$

Schwaab *et al*.^20,23^ had shown the existence of strong correlation of kinetic parameters in mathematical models containing more than one temperature-dependent kinetic constant. Such correlation makes the task of precise estimation of different kinetic parameters more onerous^24,25^. In such cases, re-parametrized form of Arrhenius equation is known to significantly reduce computational effort by reducing the correlation between parameters^26^. Hence, temperature-dependency of various kinetic rate coefficients (k) and adsorption equilibrium constants (K) in our proposed model was determined using the re-parametrized form of Arrhenius equation (Eq. (8)). In Eq. (8), *k*_*i*_ refers to the rate coefficient of reaction *i*, *ki*,_0_ is the constant related to frequency coefficient, *k*_*i*,*Ea*_ is the constant related to activation energy of a reaction, *R* is universal gas constant (8.314 J/mol. K), and *T* is temperature (in Kelvin).

8 $${k}_{i}=\exp [{k}_{i,0}-\frac{{k}_{i,{E}_{a}}}{R}(\frac{1}{T}-\frac{1}{323})]$$

## Parameter Estimation and Model Validation

To determine the various rate constants, experimentally determined [*D*] and [*E*] values were used as input parameters in our proposed model for all four scenarios (S1, S2, S3 and S4). First-order differential equations (Eqs (1–4)) were integrated using Forward-Euler method with a step size of 1 s. Genetic algorithm was used to minimize the cost function (*J*) (Eq. (9)) by running 1000 iterations that had the same boundary conditions for all kinetic parameters in all scenarios. Initially, various rate constants were determined for the epoxidation reaction at 40, 50 and 60 °C for all four scenarios.

9 $$J=\sqrt{\frac{1}{n}{\sum }^{}(\frac{{({[D]}_{exp}-{[D]}_{model})}^{2}}{{{[D]}_{exp,max}}^{2}})+\frac{1}{n}\sum (\frac{{({[E]}_{exp}-{[E]}_{model})}^{2}}{{{[E]}_{exp,max}}^{2}})}$$

where [*D*]_*exp*_ = IV/(2*126.9), mol/100 g of oil and [*E*]_*exp*_ = EV/16, mol/100 g of oil, and n = 8.

To estimate the robustness of our proposed model and obtain a clear understanding of the influence of double bond position on its reactivity, all scenarios were validated. While existing epoxidation kinetics models described in literature^6,9,11,14,15^ does not focus on model validation step, this work demonstrates a new method to validate the epoxidation kinetics model. Such validation of model will not only determine the robustness of the model, but also enhance their suitability for understanding the reaction kinetics of epoxidized triglycerides that possess varying amount of unsaturated fatty acid composition.

Under the model validation process, initially, the model predicted rate constants at 40 and 60 °C for various reactions (in each scenario) were used to obtain the pre-exponential factor and activation energy of the generalized Arrhenius equation (Eq. (10)). From the calculated pre-exponential factor and activation energy, rate constants at 50 °C were then calculated using Eq. (10).

10 $$k=A{e}^{-\frac{{E}_{a}}{RT}}\ldots \ldots $$

where *k* – rate constant, A – pre-exponential factor and E_a_ – activation energy.

This pre-calculated rate constants were used as input parameters and the model was allowed to predict [*D*] and [*E*] values, which were then compared with their corresponding experimentally determined values.

Error percentage (%) between experimentally determined and model-predicted iodine value (IV) and epoxy value (EV) was calculated using Eqs (11) and (12).

11 $$ \% D\,Error=\frac{RMS\,({D}_{exp}-{D}_{model})\times 100}{Mean({D}_{exp})}\ldots \mathrm{..}$$

12 $$ \% E\,Error=\frac{RMS\,({E}_{exp}-{E}_{model})\times 100}{Mean({E}_{exp})}\ldots \mathrm{..}$$

## Results and Discussion

Figure 1b shows the chemical composition of perilla oil^27,28^. While the average molecular weight of triglyceride (perilla oil) was estimated to be ~871 g/mol, its double bond functionality (i.e., average no. of double bonds per mole of triglyceride) was calculated as ~7. Iodine value of perilla oil was experimentally determined to be 196.6 g/100 g of oil, (i.e., 0.775 mol/100 g of oil)^3^.

### Influence of double bond position on its reactivity

**Figure 2:**
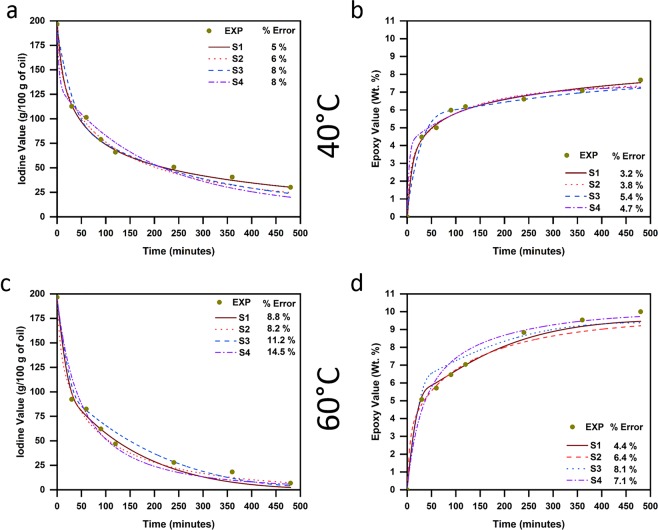
Experimentally obtained and model-predicted (for all four scenarios) during parameter estimation: (**a**) Iodine values and (**b**) Epoxy values at reaction temperature of 40 °C; (**c**) Iodine values and (**d**) Epoxy values at reaction temperature of 60 °C.

**Figure 3:**
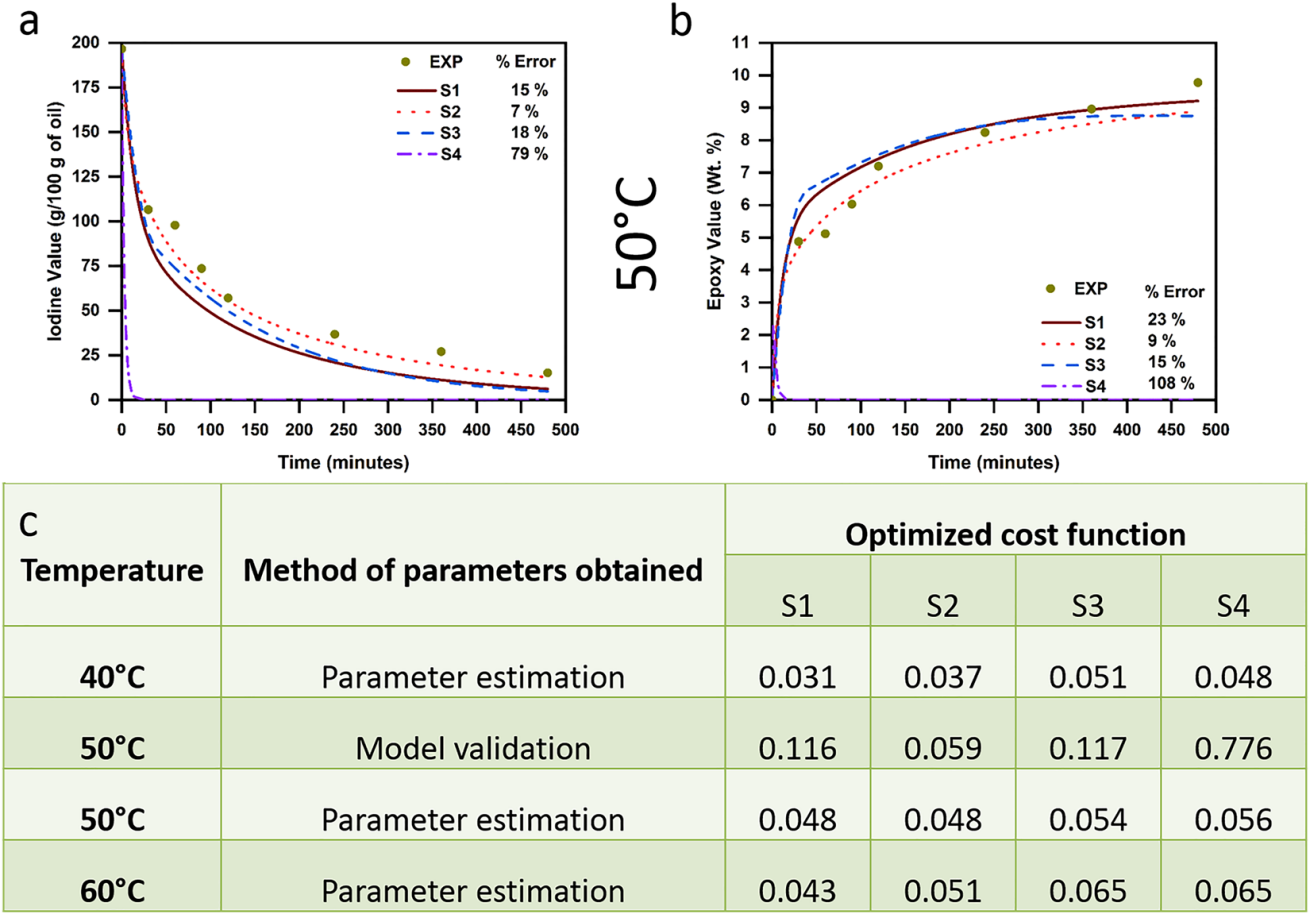
Experimentally obtained and model-predicted (for all four scenarios): (**a**) Iodine values and (**b**) Epoxy values during model validation at reaction temperature of 50 °C; and (**c**) Optimized cost function (RMS value) of the developed model for all four scenarios at the three reaction temperatures during parameter estimation and for 50 °C during model validation.

Experimentally determined (shown as points) and model-predicted (shown as lines for all scenarios) IV and EV, along with their respective error % (using Eqs (11) and (12)) are shown for 40 °C (Fig. 2a,b) and 60 °C (Fig. 2c,d) respectively. Further, Fig. 3a,b show IV and EV predicted at 50 °C during the validation of our proposed model, while Fig. 3c gives the values of the optimized cost function at all reaction temperatures obtained during parameter estimation and model validation (at 50 °C) for all four scenarios.

As can be seen (Figs 2a–d and 3c), both error % and optimized cost function obtained during parameter estimation were observed to be higher for scenarios S3 and S4 at all three temperatures. In contrast, scenarios S1 and S2 showed the least error and cost function values, with S1 predicting experimental observations more accurately when compared to S2 during parameter estimation. However, upon the validation of our model (Fig. 3a,b), scenario S2 was observed to predict experimental observations accurately with least error (<10%) and cost function (0.06). Conversely, scenario S4 exhibited very high error (>50%) and cost function (0.776), thereby failing completely in validating itself as a likely scenario, while scenarios S1 and S3 had higher error (≥15%) compared to S2, even though their predicted values were close to those observed experimentally when compared to S4.

Based on these observations (from Figs 2a–d and 3a–c), our model indicates that the assumptions made in scenario S2 are the most likely to explain experimentally observed epoxidation behavior. In other words, the reactivity of double bonds at the 9^th^ and 12^th^ positions are the same, while that of double bond at the 15^th^ position is different. This is in line with similar findings reported earlier by Scala and Wool^5^. They attributed this to the high influence of steric and electronic effects of glycerol center on double bonds closer to them (i.e., 9^th^ and 12^th^ positions), with no influence of such effects on double bonds that are farther from the glycerol center (i.e., 15^th^ position).

Conventionally, epoxidation kinetics of various triglycerides has been modelled using scenario S4 in existing literature^6,9,11,14,15,17^, indicating that models based on this scenario are highly robust and accurately predict epoxidation of different triglycerides. However, our model indicates that scenario S4 is the least likely to explain experimentally observed epoxidation behavior of high-linolenic perilla oil. This stark contrast is due to the absence of linolenic acid (C18:3) – that possesses double bond in the 15^th^ position – in triglycerides used in aforementioned studies, while perilla oil (used in this study) has high linolenic content (>50%). This means that scenarios S2 (the most accurate) and S4 (the least accurate) in our study are same for high-oleic triglycerides that have been studied till date.

Together, these explanations highlight the vital need for developing a model that robustly captures the variation in reactivity of double bonds for synthesizing EVOs with higher oxirane content – an aspect achieved by our model. In addition to capturing this variation, our model also gives results that provide further insights into their epoxidation behavior. An explanation of some such insights for two scenarios (S1 and S2) is provided henceforth.

### Reactivity of double bond and epoxy groups at different bond positions – Scenario S1

**Figure 4:**
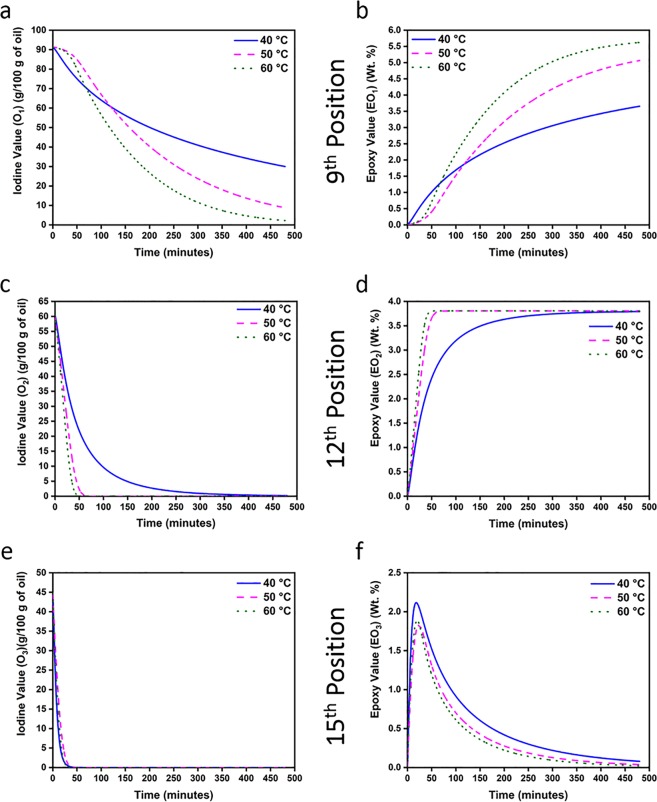
(**a**–**f**) Variation in the reactivity of the double bond and epoxy groups, based on their position at different reaction temperatures and reaction durations, for scenario S1.

Figure 4a–f shows the variation in double bond and epoxy concentration – based on their bond position – as predicted by our proposed model for scenario S1 at all three reaction temperatures. Figure 4a indicates sluggish reactivity of the double bond present at the 9^th^ position. Nevertheless, almost all double bonds at this position are predicted to participate in epoxidation reaction at both 50 and 60 °C. On the other hand, significant amount of residual double bond content is predicted to exist at 40 °C, indicating that the epoxidation reaction is incomplete even after 8 h. Conversely, Fig. 4c,e indicate high reactivity of double bonds present at the 12^th^ and 15^th^ positions at reaction temperatures of 50 and 60 °C, showing that they get consumed within the first 50 minutes of the reaction. However, at 40 °C, double bond at the 12^th^ position is predicted to react slowly when compared to the double bond at the 15^th^ position.

Contrastingly, the reactivity of epoxy group – which indicates its stability or the probability of cleavage of epoxy groups – is shown in Fig. 4b,d,f (corresponding to the 9^th^, 12^th^ and 15^th^ positions respectively). As can be seen, at all reaction temperatures, epoxy groups formed at the 15^th^ position (Fig. 4f) are predicted by our proposed model to have undergone cleavage. However, epoxy groups formed at the 9^th^ and 12^th^ positions (Fig. 4b,d) are predicted to have not participated in the ring-opening reaction.

Nonetheless, as mentioned earlier, scenario S1 shows higher error when compared to S2 during model validation (at 50 °C). Also, as can be seen from Tables 1 and 2, the confidence interval of estimated kinetic rate constants and equilibrium constants is significantly higher for S1 than for S2. These two aspects indicate that scenario S1 is not the most accurate scenario for explaining experimentally observed epoxidation behavior. Hence, variation in the reactivity of double bond and epoxy groups at different positions, as predicted in scenario S2, is provided in the subsequent section.

**Table 1:** Kinetic rate constants for epoxidation and ring-opening reactions, as determined by fitting experimentally obtained values at 40, 50 and 60 °C to our proposed model for scenario S1.

Parameter	40 °C – S1 (Parameter Estimation)	50 °C – S1 (Model Validation)	60 °C – S1 (Parameter Estimation)
k_2a_	0.0027 ± 0.00173	0.0064	0.0163 ± 0.0016
k_2b_	0.04358 ± 0.06772	0.1006	0.9040 ± 0.1787
k_2c_	0.03095 ± 0.02438	0.3822	2.0971 ± 0.1598
k_3a_	3.9E-05 ± 4E-05	6.1E-08	5.3E-11 ± 5E-11
k_3b_	9E-06 ± 8.5E-06	4.8E-11	8.3E-12 ± 6.6E-12
k_3c_	0.0045 ± 0.002	0.0036	0.0036 ± 0.0007
K_a_	0.0177 ± 0.0125	0.0571	0.2268 ± 0.1120
K_f_	375.95 ± 410.621	2.849E04	195557 ± 291197
K_h_	17.6172 ± 1.2377	1.3834	0.1835 ± 0.2464
K_p_	9751.88 ± 16834.80	130.45	5380180 ± 5017733
K_w_	32.2798 ± 23.0878	219.56	1472.21 ± 482.25

**Table 2:** Kinetic rate constants for epoxidation and ring-opening reactions, as determined by fitting experimentally obtained values at 40, 50 and 60 °C to our proposed model for scenario S2 and the activation energies of various reactions.

Parameter	40 °C – S2 (Parameter Estimation)	50 °C – S2 (Model Validation)	60 °C – S2 (Parameter Estimation)	Activation energy (kJ/mol)
k_2d_	0.0025 ± 0.0002	0.0040	0.0062 ± 2.5E-5	39.35
k_2c_	0.0515 ± 0.0075	0.0557	0.1170 ± 0.0036	25.92
k_3d_	2.1E-4 ± 2E-5	5.26E-08	3.1E-11 ± 1.1E-12	−688.40
k_3c_	0.0021 ± 0.0003	0.0038	0.0047 ± 0.0003	44.90
K_a_	0.0393 ± 0.0190	0.1559	0.3568 ± 0.0189	
K_f_	530.19 ± 986.52	113.894	11505.70 ± 1495.45	
K_h_	17.173 ± 18.601	1.8827	0.0534 ± 0.0647	
K_p_	5.0110 ± 4.7342	38.648	296.547 ± 118.557	
K_w_	6.0272 ± 6.3088	60.968	380.304 ± 176.274	

### Reactivity of double bond and epoxy groups at different bond positions – Scenario S2

**Figure 5:**
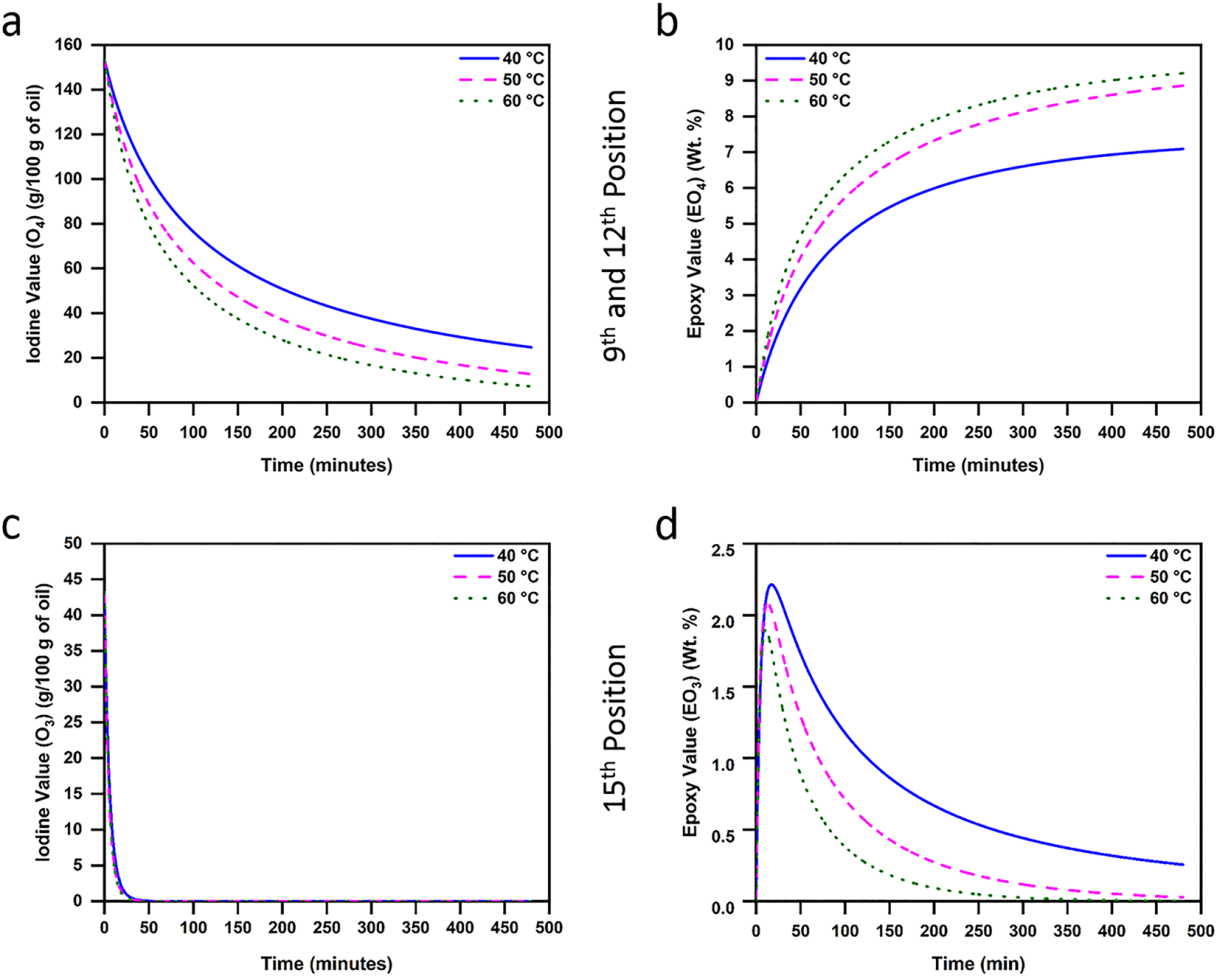
(**a**–**d**) Variation in the reactivity of the double bond and epoxy groups, based on their position at different reaction temperatures and reaction durations, for scenario S2.

Figure 5a–d highlight the variation in double bond and epoxy concentration with increasing reaction duration at all three reaction temperatures, as predicted by scenario S2. Further, Table 2 provides the various kinetic rate constants (k) and equilibrium constants (K) as predicted by our model and shows that the confidence interval for various rate constants (k) was observed to be within 90% for 40 °C and 95% for 60 °C, indicating the robustness of scenario S2 in our proposed model.

As can be seen (Fig. 5a), double bonds at the 9^th^ and 12^th^ positions exhibit sluggish reactivity at 40 °C, resulting in a significant number of un-reacted double bonds. However, the rate of reactivity of double bonds at these positions showed increase with increase in temperature to 50 and 60 °C. At the same time, Fig. 5b indicates that epoxy groups formed at the 9^th^ and 12^th^ positions did not undergo cleavage. These observations are comparable to the predictions made for variation in epoxy value (EV) in scenario S1 (Fig. 4b,d).

On the other hand, Fig. 5c indicates that double bond at the 15^th^ position reacts completely within the first 50 minutes of epoxidation reaction, while the epoxy group formed at this position is prone to undergo cleavage (Fig. 5d). Hence, for both double bond and epoxy group at the 15^th^ position, rate of reactivity is comparable across both scenarios S1 (Fig. 4e,f) and S2 (Fig. 5c,d). This is a divergent outcome from that observed in most studies on reaction kinetics of EVOs till date, which show significant epoxy cleavage (at 9^th^ and/or 12^th^ positions) in oleic-rich vegetable oils^17,29–33^. Hence, the behavior (predicted by our model) of epoxy groups present at the 9^th^ and 12^th^ positions – of not undergoing oxirane cleavage via attack by formic acid – could be due to the combination of three factors.

The first factor is the possible steric effect of the hydroxylated group present at the 15^th^ position (formed due to oxirane cleavage) on incoming formic acid. This effect is hypothesized to prevent any interaction between formic acid and the epoxy groups present at the 9^th^ and 12^th^ positions, thereby preventing their cleavage.

The second contributing factor is the combination of steric and electronic effects as reported by Scala and Wool^34^ in their study on reaction kinetics of acylation reaction (i.e., cleavage of epoxy groups and formation of acrylic group) of EVOs. They observed that oleic-rich vegetable oils exhibited higher reaction rates for acylation reaction when compared to oils containing higher amount of linoleic (C18:2) and linolenic acid (C18:3) groups, a finding they attributed to steric and electronic effects. Steric effect was generated by the presence of multiple epoxy groups in the same fatty acid chain that hindered other chemical species from attacking these groups. Simultaneously, electronic effect generated due to the glycerol center was considered responsible for preventing the cleavage of epoxy group present at the 9^th^ position in fatty acids.

A third interesting factor that explains the low levels of oxirane cleavage, as observed via decrease in kinetic rate constant for oxirane cleavage (at 9^th^ and 12^th^ positions) with increase in reaction temperature (Tables 1 and 2), is the degradation of formic acid at higher temperatures due to higher reactivity of hydrogen peroxide^35,36^. Such degradation is likely to reduce the possibility of oxirane cleavage via attack by formic acid on epoxy groups, especially for epoxy groups at relatively less-accessible positions (i.e., 9^th^ and 12^th^ positions).

### Activation energy of various reactions

Activation energies of epoxy formation and cleavage reactions at various double bond positions (9^th^, 12^th^ and 15^th^) are provided in Table 2. These activation energies were calculated for scenario S2, since this scenario is observed to be the most accurate predictor of epoxidation kinetics in this study (as explained earlier). As can be seen, barring the activation energy for oxirane cleavage at the 9^th^ and 12^th^ positions (k_3d_), all other activation energy values are positive and are in line with corresponding values for epoxidation of other vegetable oils^3^. In contrast, epoxy cleavage at the 9^th^ and 12^th^ positions exhibits a negative activation energy (−688.40 kJ/mol) - the mathematical result of decrease in rate constant (k_3d_) with increase in temperature. While such negative activation energy is uncommon, it is explained by two factors that together inhibit oxirane cleavage at the 9^th^ and 12^th^ positions: (a) Increasing oxirane cleavage at 15^th^ position with increase in temperature, which enhances the steric effect of hydroxylated group at the 15^th^ position on incoming formic acid; and (b) Degradation of formic acid at higher temperatures. Hence, the reaction becomes more difficult and less likely to occur at higher temperatures, leading to the obtainment of negligible rate constant at higher temperatures.

## Conclusion

A pseudo two-phase model that captures the variation in reactivity of chemical groups based on their position was developed to study epoxidation kinetics of high-linolenic perilla oil. Four different scenarios were considered to understand the reactivity of chemical species at different positions in the triglyceride molecule. The results clearly indicate that chemical groups at the 9^th^ and 12^th^ positions possess the same reactivity and is significantly different from that of the same group at the 15^th^ position. The robustness of our model was validated by calculating kinetic parameters and predicting iodine and epoxy values of epoxidized perilla oil at 50 °C.

## Methods

### Materials

Triglycerides, sourced from *Perilla frutescens*, were purchased from Dr. Adorable Inc., Chicago, IL, USA. All other chemicals required for the epoxidation reaction and subsequent characterization, such as formic acid (HCOOH), Amberlite IR 120 (particle size: 620–830 µm), 30% (w/w) hydrogen peroxide (H_2_O_2_), Wijs solution (ICl), starch, potassium iodide (KI), tetraethyl ammonium bromide (TEAB or C_8_H_20_NBr), crystal violet indicator, 0.1 N perchloric acid reagent (HClO_4_), and 0.1 N sodium thiosulfate (Na_2_S_2_O_3_), were obtained from Sigma Aldrich, WI, USA. Glacial acetic acid (CH_3_COOH) was purchased from VWR Chemicals, GA, USA. All chemicals were used in as-received state.

### Epoxidation of Perilla Oil

Perilla oil, containing >90% unsaturated fatty acids (chemically modifiable group), was epoxidized via Prilezhaev reaction, using formic acid (HCOOH) as the oxygen carrier and hydrogen peroxide (H_2_O_2_) as the oxygen donor, in the presence of acidic ion-exchange resin – Amberlite IR I20 – as the solid catalyst. The ratio between moles of unsaturation (double bonds), formic acid and hydrogen peroxide was maintained at 1: 0.5: 1.5. Amberlite IR 120 was taken at 20 wt. % of perilla oil, and epoxidation reaction was carried out at three different temperatures – 40, 50 and 60 °C – for 8 h each.

Both perilla oil and the solid catalyst (AIER) were initially placed in a 1000 ml three-neck round-bottom flask and equilibrated to synthesis temperature, while being simultaneously stirred at 500 rpm (using a mechanical stirrer) for 30 min. Subsequently, HCOOH was added, and after 30 minutes, 30% (w/w) H_2_O_2_ solution was added drop-wise to this solution under constant stirring. System temperature was maintained below 110 °C for each set of experiments throughout the entire duration of epoxidation. In each experiment, aliquot samples were removed from the solution at intervals of 30 min for the first two hours (i.e., at 0.5, 1, 1.5 and 2 h), followed by further removal at intervals of 2 h for the next six hours (i.e., at 4, 6 and 8 h) to monitor the progress of the epoxidation reaction. Extracted epoxidized perilla oil (EPeO) was dissolved in 2 ml of toluene and subsequently subjected to several cycles of washing with distilled water, until the aqueous phase showed pH of 7. The washed EPeO was dried using anhydrous sodium sulfate (Na_2_SO_4_) to remove any small traces of moisture and vacuum-dried at 40 °C to ensure the complete evaporation of any solvent present.

### Characterization

#### Iodine Value

Iodine value of a substance refers to the mass of iodine (I_2_, in grams) consumed by 100 g of that substance, and is calculated to determine the extent of unsaturation (i.e., moles of double bonds) in fatty acids^37^. In this work, iodine values of both perilla oil and EPeO (withdrawn at different time intervals) were determined as per ASTM D5768 standard to understand the extent of opening of double bonds.

#### Epoxy Value (Oxirane Oxygen Content)

Epoxy value of EPeO (withdrawn at different time intervals) was determined as per ASTM D1652-11 standard to study the progression of epoxidation reaction. Epoxy value (*E*) in EPeO was calculated using Eq. (13), where *V* is the volume of HClO_4_ required for titration (ml), *N* is the normality of HClO_4_ (0.1 N), and *W* is the mass of EPeO used for titration (g).

13 $$E=4.3\times V\times (\frac{N}{W})$$

## Data Availability

The datasets generated during and/or analyzed during the current study are available from the corresponding author on reasonable request.
